# A meta-analysis: Is there any association between MiR-608 rs4919510 polymorphism and breast cancer risks?

**DOI:** 10.1371/journal.pone.0183012

**Published:** 2017-08-22

**Authors:** Jing Wang, Xiangyi Kong, Zeyu Xing, Xiangyu Wang, Jie Zhai, Yi Fang, Jidong Gao

**Affiliations:** Department of Breast Surgical Oncology, China National Cancer Center/Cancer Hospital, Chinese Academy of Medical Sciences and Peking Union Medical College, Chaoyangqu, Panjiayuan, Beijing, P. R. China; University of Birmingham, UNITED KINGDOM

## Abstract

**Object:**

To combine the data from previously conducted studies about the associations between miR-608 rs4919510 polymorphism (C>G) and breast cancer risks.

**Methods:**

According to the Preferred Reporting Items for Systematic Reviews and Meta-Analyses (PRISMA) guidelines, we conducted a systematic review of the related literatures searched from PubMed, Embase, Cochrane Library, Web of Science, and China National Knowledge Internet (CNKI) (time: ~ December 2016). Using DerSimonian-Laird random-effects models [Pooling Model: Mantel Haenszel (MH)], odd ratios (ORs) with 95% confidence intervals (95% CIs) were estimated in the allele model, homozygote model, heterozygote model, dominant model and recessive model. Heterogeneity was analyzed using Labbr plots and I^2^ statistic. Publication bias was analyzed using contour-enhanced funnel plots.

**Results:**

We included 5 eligible studies with 7948 patients. The ORs and their 95% CIs in the 5 genetic models mentioned above were 1.009 (95% CI: 0.922, 1.104; p = 0.847), 1.098 (95% CI: 0.954, 1.264; p = 0.194), 1.076 (95% CI: 0.956, 1.211; p = 0.227), 1.043 (95% CI: 0.880, 1.236; p = 0.628), 1.007 (95% CI: 0.906, 1.118; p = 0.899), respectively.

**Conclusion:**

In the present meta-analysis, no relationships between miR-608 rs4919510 polymorphism (C>G) and the risk of breast cancer were found. More studies are warranted to further validate the conclusion.

## Introduction

As one of the malignancies with the highest incidence and mortality rates globally, breast cancer accounts for more than a million cases every year[1, 2]. In North America, the incidence of breast cancer is the highest among women, and its mortality ranks the second in all cancer deaths in female[1]. Although causes of breast cancer are not yet completely understood, genetic factors are considered to play pivotal roles in the pathogenesis of this malignancy[3].

MicroRNAs, as gene expression regulators, regulate a variety of biochemical processes, such as apoptosis, proliferation, metabolism, cellular differentiation, and cancer development[3–6]. It has been proposed that rs4919510 C>G variant in miR-608 can alter its binding to target genes. MiR-608 expectedly targets growth hormone receptor (GHR), interleukin-1 alpha (IL1A), insulin receptor (INSR), and TP53[7–9]. Several studies examined the impacts of miR-608 rs4919510 C>G on breast cancers risks, but the results were inconsistent[8, 10–13]. In Huang's report, the results showed that the single nucleotide polymorphism (SNP) could alter the secondary structure of primary miR-608[11]. A single nucleotide variation located at introns has also been experimentally shown to change DNA and RNA secondary structures, and consequently associate with gene expressions and diseases. A single study might not be able to conclusively confirm the correlations, especially if the study is of small-sample-size. In 2013, Hu et al. conducted a meta-analysis regarding 8 precursor-miRNA SNPs (including miR-608 rs4919510) in 8 common cancers (including breast cancer), and did not find significant associations between miR-608 and cancers[14]. However, in their study, the authors generally analyzed all caner types together instead of specifically discussing breast cancer. Besides, the studies included in their study were limited and several more recent studies have been finished up to now. To further elucidate the exact effects of miR-608 rs4919510 polymorphism on breast cancer risk, we accumulate data from different case control studies and perform this meta-analysis to make an evaluation.

## Methods

### Publication search and selection criteria

Two authors (XK and JW) independently searched the database of Embase, PubMed, Cochrane Library, Chinese National Knowledge Infrastructure (CNKI), and Web of Knowledge (time: ~ December 2016). Search terms used separately or in combination were: “breast carcinoma or breast cancer” and “rs4919510 or miR-608” and “mutation or variant or polymorphism”. Detailed searching strategies with the start and end date of searches were listed in S1 Table. For example, for PubMed database, the search strategy “breast[Title/Abstract] AND ((MiR-608[Title/Abstract] OR MicroRNA-608[Title/Abstract]) OR rs4919510[Title/Abstract])” was adopted and 7 articles were obtained. We reviewed related references to find out other potentially eligible studies. The exclusion criteria and inclusion criteria are listed in Table 1.

**Table 1:** Inclusion criteria for study selection in this meta-analysis.

**Number**	**Inclusion criteria**
1	Case-control studies.
2	The studies evaluated the associations between miR-608 rs4919510 polymorphism and breast cancer risk.
3	The studies included detailed genotyping data (total number of cases and controls, number of cases and controls with C/C, C/G, and G/G genotypes).
4	Studies focusing on human being.
**Number**	**Exclusion criteria**
1	The design of the experiments was not case-control.
2	The source of cases and controls, and other essential information were not provided.
3	The genotype distribution of the control population was not in accordance with the Hardy—Weinberg equilibrium (HWE).
4	Reviews and duplicated publications.

### Data extraction

According to the inclusion criteria set in Table 1, 2 independent authors (JW and XK) reviewed and extracted the needed data and information from the included articles. The following data were extracted: author name, publication year, country, ethnicity or race (Asian, Caucasian or others), genotyping methods, total number of controls and cases, number of controls and cases with rs4919510 polymorphism, number of cases and controls with C/C, C/G, and G/G genotypes, control source (hospital-based or population-based), and P value for Hardy-Weinberg equilibrium (HWE).

### Methodological quality assessment

According to the methodological quality assessment scale (see Table 2), which was adjusted from a previous publication by Guo et al. in PLos One in 2012[15], two authors (XK and JW) independently estimated the quality of the included studies. Disagreement would be solved by discussion. In this methodological quality assessment scale, 5 items, including quality control of genotyping methods, source of controls, sample size, cases representativeness and HWE were carefully checked. The quality scores range from 0 to 10. The higher the score is, the higher the quality of the study.

**Table 2:** Scale for methodological quality assessment.

Criteria	Score
**1. Representativeness of cases**	
Breast cancer diagnosed according to acknowledged criteria.	2
Mentioned the diagnosed criteria but not specifically described.	1
Not Mentioned.	0
**2. Source of controls**	
Population or community based	3
Hospital-based Breast-cancer -free controls	2
Healthy volunteers without total description	1
Breast-cancer -free controls with related diseases	0.5
Not described	0
**3. Sample size**	
>300	2
200–300	1
<200	0
**4. Quality control of genotyping methods**	
Repetition of partial/total tested samples with a different method	2
Repetition of partial/total tested samples with the same method	1
Not described	0
**5. Hardy-Weinberg equilibrium (HWE)**	
Hardy-Weinberg equilibrium in control subjects	1
Hardy-Weinberg disequilibrium in control subjects	0

### Statistical analysis

Our study was based on the PRISMA checklists (S2 Table) and the meta-analysis-on-genetic-association-studies-form (S3 Table)[16]. HWE in each study was assessed, followed by the calculations of ORs with 95% CIs to reflect the correlation strength between rs4919510 polymorphism and the risk of breast cancer. The pooled ORs were calculated and used for comparisons respectively in allele model (G vs. C), homozygote model (GG vs. CC), heterozygote model (CG vs. CC), dominant model (CG+GG vs. CC), and recessive model (GG vs. CC+CG). The Labbe plot, Cochran's Q-test, and I^2^ statistic (Table 3) were used to access the heterogeneities [17]. Since fixed effect models might underperform in the presence of any heterogeneity[18], while DerSimonian-Laird random-effects models are more conservative and able to provide better estimates with wider confidence intervals, we adopted the latter [Pooling Model: Mantel Haenszel (MH)] for all the analyses of all the 5 genetic models[18]. To estimate the stabilities of the pooled results, probabilistic sensitivity analyses of meta-analysis (explanation in Table 3) were made[19]. By contour-enhanced funnel plots (explanation in Table 3), we accessed possible publication biases.

**Table 3:** The statistical methods used in this meta-analysis and their explanation.

Statistic means	Goals and Usages	Explanation
Labbe plot	To evaluate heterogeneity between the included studies	In Labbe Figure, if the points basically present as a linear distribution, it can be taken as an evidence of homogeneity.
Cochran’s Q test	To evaluate heterogeneity between the included studies	Cochran's Q test is an extension to the McNemar test for related samples that provides a method for testing for differences between three or more matched sets of frequencies or proportions. Heterogeneity was also considered significant if P < 0.05 using the Cochran's Q test.
I^2^ index test	To evaluate heterogeneity between the included studies	The I^2^ index measures the extent of true heterogeneity dividing the difference between the result of the Q test and its degrees of freedom (k– 1) by the Q value itself, and multiplied by 100. I^2^ values of 25%, 50% and 75% were used as evidence of low, moderate and high heterogeneity, respectively.
Sensitivity analysis	To examine the stability of the pooled results	A sensitivity analysis was performed using the one-at-a-time method, which involved omitting one study at a time and repeating the meta-analysis. If the omission of one study significantly changed the result, it implied that the result was sensitive to the studies included.
Contour-enhanced funnel plot	Publication bias test	Visual inspection of the Contour-enhanced funnel plots was used to assess potential publication bias. Asymmetry in the plots, which may be due to studies missing on the left-hand side of the plot that represents low statistical significance, suggested publication bias. If studies were missing in the high statistical significance areas (on the right-hand side of the plot), the funnel asymmetry was not considered to be due to publication bias

P < 0.05 reflected statistical significance. The statistical analyses were made by Stata 13.0 (StataCorp LP, College Station, TX, USA) software. The Stata commands is metan.

## Results

### Search results and characteristics of the studies

**Fig 1:**
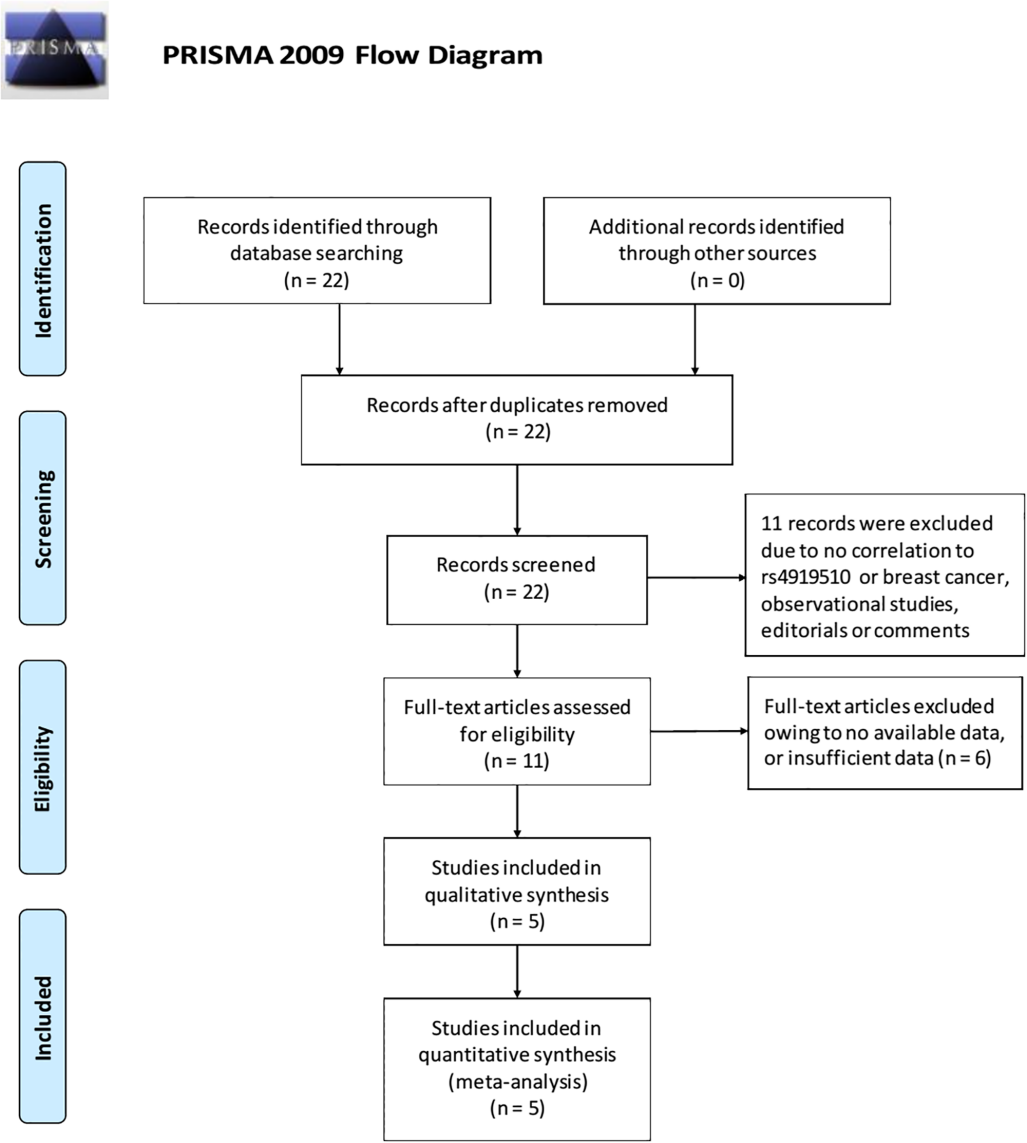
Literature search and selection of articles. *From*: Moher D, Liberati A, Tetzlaff J, Altman DG, The PRISMA Group (2009). *P*referred *R*eporting *I*tems for *S*ystematic Review s and *M*eta-*A*nalyses: The PRISMA Statement. PLOS Med 6(6):e1000097. Doi:10.1371/journal.pmed1000097. **For more information, visit** www.prisma-statement.org.

According to PRISMA statement, a study selection flowchart was reported in Fig 1.

A total of 35 studies were identified: 7 in Pubmed, 7 in Embase, 0 in Cochrane Library, 14 in Web of Science and 7 in CNKI (S1 Table). Finally, a total of 5 articles involving 7948 patients were included[8, 10, 11, 13, 20]. Two studies were on the basis of Caucasian backgrounds and were done in Iran (352 cases) and Chile (1247 cases)[20]. Three studies were on the basis of Asian backgrounds and were done in China (6349 cases in total)[10, 11, 13]. Four studies were written in English[8, 10, 11, 20] and 1 was in Chinese[13]. Breast cancers were all confirmed by histopathologic examinations. In all included studies, genotype distributions of rs4919510 (C > G) in the controls were consistent with HWE. A variety of genotyping methods were applied including SNPstream[11, 13], PCR-RFLP[8], TaqMan Genotyping Assay[20] and Sequenom MassARRAY RS100[10]. Genomic miRNA was isolated from blood samples in all included studies. Controls were matched in terms of age. Four studies were population-based[8, 11, 13, 20] and 1 was hospital-based[10]. Excluded studies and the rational for the exclusion were listed in Table 4. The characteristics including the basic information of the literatures, the original data, P for HWE, and the methodological quality assessment results of the included literatures were shown in Table 5.

**Table 4:** Excluded studies and the rational for exclusion.

Excluded studies	Rational for exclusions
Jeyapalan et al. (2011)	This study only explored the targets of miR-608 and its interactions with CD44 and CDC42 3’-UTRs.
Huang et al. (2012)	Related data could not be extracted from the results.
Hu et al. (2013)	This is a meta-analysis that lacks original data.
Jiao et al. (2014)	This study only provided the data of associations between miR-608 and survival in breast cancer patients.
Rah et al. (2015)	This study mainly focuses on the relationships between miR0608 and primary ovarian insufficiency (POI) risks.
Ma et al. (2015)	Related data could not be extracted from the results.

**Table 5:** Characteristics of studies included in the meta-analysis.

Author	Year	Country	Ethnicity	Cancer type	Genotyping	Source of controls	Cases (n)				Controls (n)				P for HWE	Quality
							Total	CC	CG	GG	Total	CC	CG	GG		
Huang et al.	2012	China	Asian	Breast cancer	SNPstream	Population-based	763	128	381	254	1417	277	684	456	0.4762	8
Shao et al.	2012	China	Asian	Breast cancer	SNPstream	Population-based	1118	192	545	381	1908	354	914	640	0.9032	7
Dai et al.	2016	China	Asian	Breast cancer	Sequenom MassARRAY RS100	Hospital-based	560	107	296	157	583	113	287	183	0.98	8
Hashemi et al.	2016	Iran	Caucasian	Breast cancer	PCR-RFLP	Population-based	160	140	20	0	192	149	43	0	0.0806	8
Morales et al.	2016	Chile	Caucasian	Breast cancer	TaqMan Genotyping Assay	Population-based	440	226	174	40	807	431	310	66	0.3322	8

### Meta-analysis results

**Fig 2:**
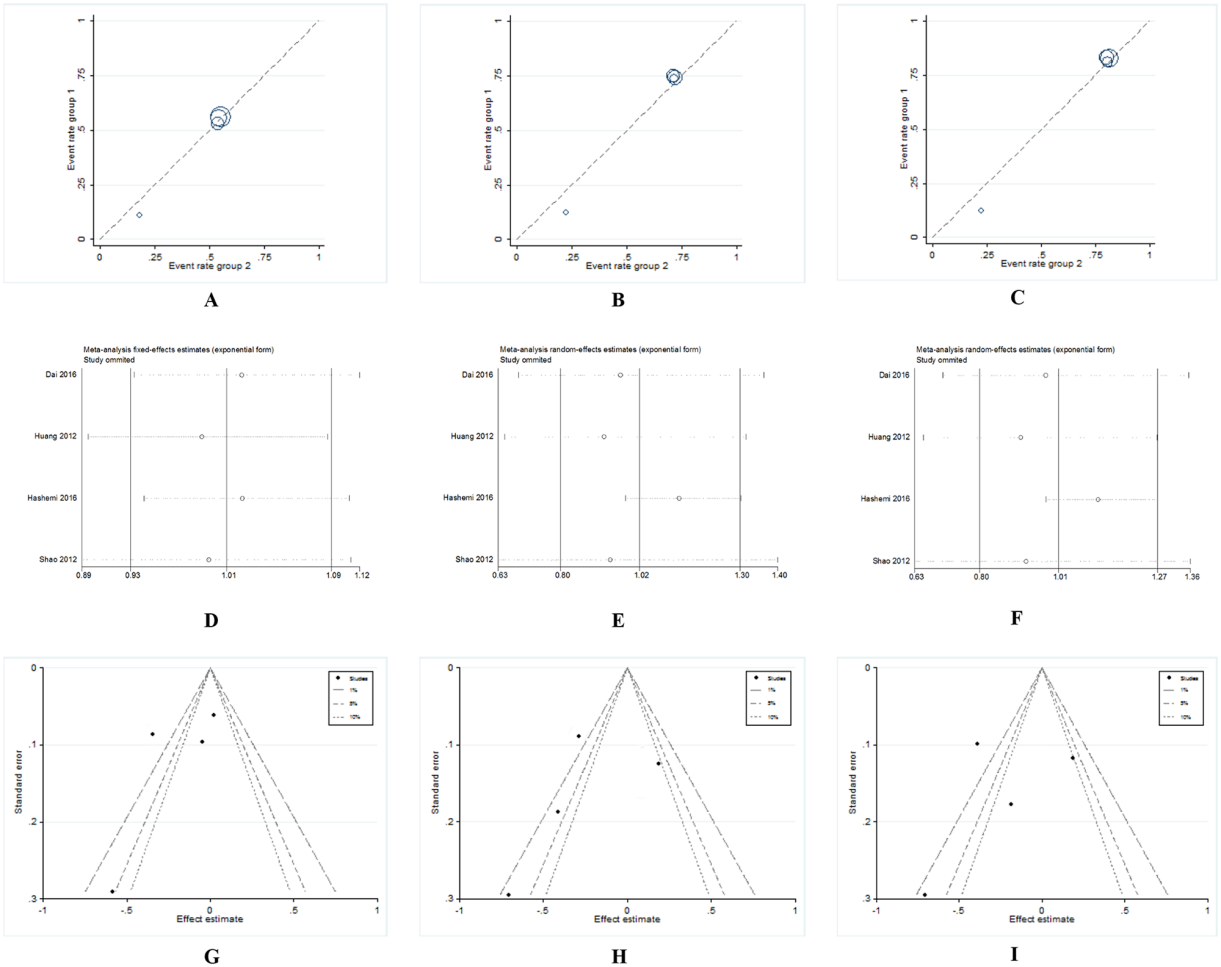
Labbe plots, sensitivity analysis plots and contour-enhanced funnel plots of the included studies focusing on the association between miR-608 rs4919510 polymorphism and breast cancer risk. Labbe plots in allele model (**A**), heterozygote model (**B**), and dominant model (**C**). Sensitivity analysis in allele model (**D**), heterozygote model (**E**), and dominant model (**F**). Contour-enhanced funnel plots in allele model (**G**), heterozygote model (**H**), and dominant model (**I**).

**Fig 3:**
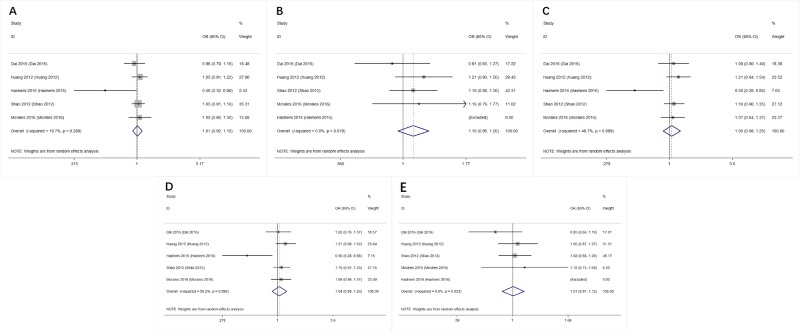
Forest plots (individual and pooled effects with 95% CI) regarding the association between miR-608 rs4919510 polymorphism and breast cancer risk. **A**: allele model, random effect model; **B**: homozygote model, random effect model; **C**: heterozygote model, random effect model; **D**: dominant model, random effect model; **E**: recessive model, random effect model.

The main results including heterogeneity tests, effect models adopted accordingly, and the pooled OR with 95% CI and P value of this meta-analysis were shown in Table 6. The Labbe plots for allele model, heterozygote model and dominant model were shown in Fig 2-A, 2B and 2C. For overall studies, there were no statistically correlations between miR-608 rs4919510 polymorphism and decreased or increased breast cancer risks in all the 5 models (allele model: OR 1.009, 95% CI 0.922, 1.104; p = 0.847; Fig 3-A; homozygote model: OR 1.098, 95% CI 0.954, 1.264; p = 0.194; Fig 3-B; heterozygote model: OR 1.076, 95% CI 0.956, 1.211; p = 0.227; Fig 3-C; dominant model: OR 1.043, 95% CI 0.880, 1.236; p = 0.628; Fig 3-D; recessive model: OR 1.007, 95% CI 0.906, 1.118; p = 0.899; Fig 3-E).

**Table 6:** The results of meta-analysis for various genotype models.

Genetic model		Heterogeneity test						Test of Association				Publication bias
Name	Explanition	Q value	d.f.	I-quared	Tau-squared	P Value	Effect model	Pooled OR	95% CI	P value	Statistical significance	
Allele model	G vs. C	4.98	4	19.7%	0.0021	0.289	Random	1.009	[0.922, 1.104]	0.847	No	No
Homozygote model	GG vs. CC	1.78	3	0.00%	0.0000	0.619	Random	1.098	[0.954, 1.264]	0.194	No	No
Heterozygote model	CG vs. CC	7.80	4	48.7%	0.0187	0.099	Random	1.076	[0.956, 1.211]	0.227	No	No
Dominant model	CG+GG vs. CC	8.04	4	50.2%	0.0179	0.090	Random	1.043	[0.880, 1.236]	0.628	No	No
Recessive model	GG vs. CC+CG	2.19	3	0.00%	0.0000	0.533	Random	1.007	[0.907, 1.118]	0.899	No	No

### Sensitivity analysis and publication bias

Sensitivity analysis demonstrated that the pooled ORs were not affected by deleting every single study (Fig 2-G, 2H, 2I, 2J and 2K). The contour-enhanced funnel plots revealed that the studies had missing areas of high statistical significance (in the right-hand side of the plot), indicating no publication bias in this study (Fig 2-L, 2M, 2N, 2O and 2P).

## Discussion

**Fig 4:**
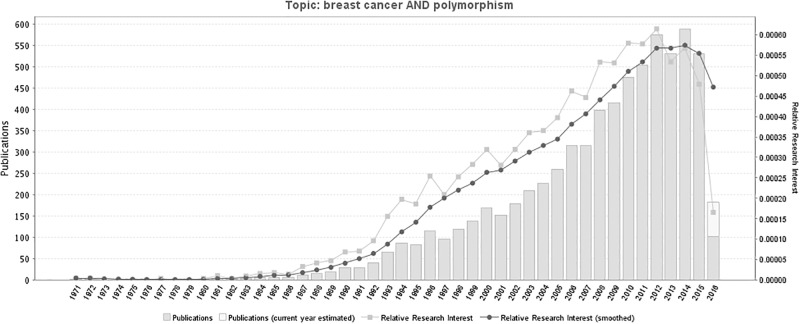
A timeline of the publications related to breast cancer-related polymorphisms. Fig 4 was generated through GoPubMed (website: http://www.gopubmed.com). GoPubMed is a knowledge-based search engine for biomedical texts. The technologies used in GoPubMed are generic and can in general be applied to any kind of texts and any kind of knowledge bases. The system was developed at the Technische Universität Dresden by Michael Schroeder and his team at Transinsight. Creation steps for this timeline: import search items to the Search Box at the home page, then click “Statistics” and download related statistical charts including the timeline and map.

Recently, gene polymorphisms which may contribute to the tumorigenesis of breast cancer have attracted more and more scholars’ attention[21]. Some genes or RNA polymorphisms have already been proposed to increase the susceptibility of breast cancers[22]. The number of studies related to breast cancer-related polymorphisms show a general tendency to increase yearly. A timeline of the literatures was shown as Fig 4, which was generated through the following website: http://www.gopubmed.com.

Recently, rs4919510 polymorphism in miR-608 has been reported to predict clinical outcomes for cancer patients in different cancer types. Hashemi et al. evaluated the impact of miR-608 rs4919510 C>G variant on the breast cancer risk[8]. They found that GC genotype decreased breast cancer risks significantly (OR = 0.49, 95% CI 0.28, 0.88; p = 0.018) compared to CC genotype. Furthermore, the G allele decreased the breast cancer risk (OR = 0.53, 95% CI 0.30, 0.92; p = 0.024)[8]. In Huang et al.’s study, miR-608 rs4919510 also affect HER2-positive breast cancer risks and tumor proliferations[11]. However, in Dai et al.’s study, for miR-608 rs4919510, no significant correlations were detected in the genetic comparison models[10]. In 2013, Hu et al. conducted a meta-analysis regarding 8 precursor-miRNA SNPs (including miR-608 rs4919510) in 8 common cancers (including breast cancer), and did not find significant associations between miR-608 and cancers[14]. However, in their study, the authors generally analyzed all caner types together instead of specifically discussing the breast cancer. Besides, the articles included in their study are limited and several more recent studies have been finished up to now.

A single study cannot be sufficient enough to confirm the correlation between miR-608 rs4919510 polymorphism and breast cancer risks convincingly, especially for small-sample-size studies. Given this, Pubmed, Embase, Cochrane Library, Web of Science, and CNKI databases were combined to further analyze the associations. The results of our study failed to demonstrate any significant correlation. This analysis is the most updated one to provide an evaluation of the correlations between miR-608 rs4919510 polymorphism and breast cancer risks.

On a contour-enhanced funnel plot, “if the area where studies are perceived to be missing are areas of high statistical significance (the right part of the funnel plot), then publication bias isn’t the cause of funnel asymmetry”[23]. In the present meta-analysis, we found no publication bias.

Several limitations existed in our study: (1) Included studies were relatively insufficient to do subgroup-analyses; (2) The effect of gene-environment interactions and gene-gene interactions was not emphasized; (3) More accurate ORs should be adjusted by patient factors such as gender, age, living styles, medication consumption and other exposure factors; (4) Only published articles were included, the unpublished and ongoing studies could convert our result; (5) When the 95% confidence intervals around I^2^ are wide, inferences about the heterogeneity extent should be cautious, thus, calculating the confidence intervals for I^2^ is important for estimating the heterogeneity if the number of included studies are large enough. However, since the present study is only a small meta-analysis, and we used DerSimonian-Laird random-effects models for all the analyses of all the 5 genetic models, we did not calculate the confidence intervals. After all, Cochran Q (i.e. chi-square) is somewhat underpowered to detect heterogeneities, especially for small meta-analyses; thus, we only used the I^2^ statistic as a rough reflection; (6) Regarding heterogeneity estimates, all these estimates are very likely off, especially for small meta-analyses, and we should be wary about homogeneity assumptions. In this smaller meta-analysis, we failed to identify any heterogeneity, which might exist. In addition, since fixed effect models might underperform in the presence of any heterogeneity, while DerSimonian-Laird random-effects models are more conservative and able to provide better estimates with wider confidence intervals, we adopted the latter for all the analyses of all the 5 genetic models; (7) Publication bias tests and plots only relevant if >10 studies are included otherwise underpowered to detect much and tend to lead to conclusions that are not justified. In the present study, we don’t have enough studies to assess, which is another limitation.

## Conclusions

In conclusion, our results suggested that miR-608 rs4919510 polymorphism may not be associated with the susceptibility of breast cancer.

## Supporting information

S1 Table Searching strategies and results for different databases. (DOC)

S2 Table PRISMA 2009 checklist. (DOC)

S3 Table meta-analysis-on-genetic-association-studies-form. (DOC)
