# Supplementary material for: A meta-analysis: Is there any association between MiR-608 rs4919510 polymorphism and breast cancer risks?
Source: PLoS One. 2017 Aug 22;12(8):e0183012. doi: 10.1371/journal.pone.0183012 (PMC5568721; doi:10.1371/journal.pone.0183012)
Supplement: S1 Table — (DOC) [file pone.0183012.s001.doc]

| **Supplemental Table S1** Searching strategies and results for different databases | | | | | | |
| --- | --- | --- | --- | --- | --- | --- |
| **Database** | **Database URL** | **Start date** | **End date** | **Search strategy** | | **Results** |
| **Pubmed** | https://www.ncbi.nlm.nih.gov/pubmed/ | Not limited | Mar 21st, 2017 | breast[Title/Abstract] AND ((MiR-608[Title/Abstract] OR MicroRNA-608[Title/Abstract]) OR rs4919510[Title/Abstract]) | | 7 |
| **Embase** | https://www.embase.com/ | Not limited | Mar 21st, 2017 | 'mir 608':ab,ti OR 'microrna 608':ab,ti OR rs4919510:ab,ti AND breast:ab,ti | | 7 |
| **Cochrane Library** | http://www.cochranelibrary.com/ | Not limited | Mar 21st, 2017 | breast and (MiR-608 OR MicroRNA-608 OR rs4919510):ti,ab,kw | | 0 |
| **Web of Science** | http://apps.webofknowledge.com/ | Not limited | Mar 21st, 2017 | # 1 TOPIC: (breast)  # 2 TOPIC: (MiR-608) OR TOPIC: (MicroRNA-608) OR TOPIC: (rs4919510)  # 1 AND # 2  Indexes=SCI-EXPANDED, SSCI, A&HCI, CPCI-S, CPCI-SSH, BKCI-S, BKCI-SSH, ESCI, CCR-EXPANDED, IC Timespan=All years | 14 | |
| **CNKI** | http://www.cnki.net/ | Not limited | Mar 21st, 2017 | 检索条件：( ( 全文=MicroRNA-608 或者 全文=rs4919510 ) 并且 主题=breast ) (模糊匹配),专辑导航：全部; 数据库：文献 跨库检索  检索方式：跨库检索  数据库：文献 | | 7 |
